# Presumed insect sting–induced Takotsubo cardiomyopathy

**DOI:** 10.1016/j.jacig.2025.100516

**Published:** 2025-06-11

**Authors:** Karl Maxemous, Noah D.H. Lewis, Jason Chung, Jumana Sarraj, Samira Jeimy

**Affiliations:** a Division of Allergy and Immunology, Department of Medicine, Western University, London, Ontario, Canada; b Department of Medicine, University of Toronto, Division of Cardiology, St Michael’s Hospital, Toronto, Ontario, Canada; c Lawson Health Research Institute, St Joseph’s Health Care London, London, Ontario, Canada

**Keywords:** Takotsubo cardiomyopathy, stress-induced cardiomyopathy, envenomation, venom immunotherapy, epinephrine, allergy, anaphylaxis

## Abstract

This report highlights a rare association between an insect sting and Takotsubo cardiomyopathy. It discusses the decisions surrounding the use and safety of epinephrine and a modified venom immunotherapy approach for desensitizing a high-risk patient with cardiovascular comorbidities.

Takotsubo cardiomyopathy (TCM) is characterized by catecholamine-induced, transient, left ventricular dysfunction that is often precipitated by acute emotional or physical stress.^1^ Insect stings can elicit reactions ranging from local skin reactions to anaphylaxis.^2^ Skin testing with venom extracts and *in vitro* testing for specific serum IgE antibodies can confirm the diagnosis of an IgE-mediated hypersensitivity reactions in the appropriate clinical context. There is a paucity of evidence in the literature regarding TCM precipitated by insect stings. Moreover, there are no clear guidelines that outline a safe and effective protocol for venom immunotherapy (VIT) in patients with TCM. In this case report, we present a patient who was presumed to have developed TCM following Hymenoptera envenomation and subsequently underwent a modified VIT protocol.

## Case

**Fig 1:**
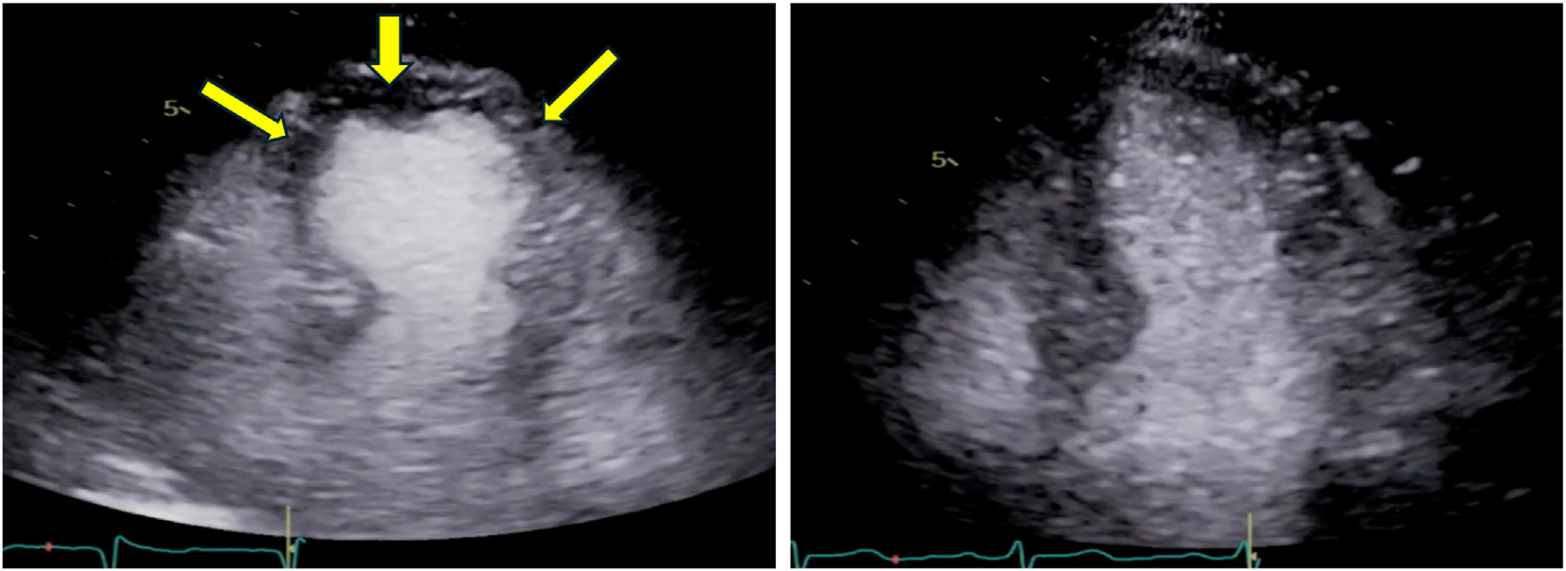
Echocardiogram demonstrating TCM. (*Leftpanel*) An apical 4-chamber view of a 2-dimensional echocardiogram of a patient with TCM with classical apical ballooning and hyperdynamic basal wall contraction of the left ventricle (*yellow arrows*). (*Right panel*) An apical 4-chamber view of a 2-dimensional echocardiogram with normal left ventricular systolic function. Clips are courtesy of the echocardiography laboratory of St Michael’s Hospital (Toronto, Ontario, Canada) and used with permission.

A healthy 68-year-old female returning from vacation developed localized cutaneous swelling and erythema after a sting by an unidentified insect. Within 24 hours, she developed generalized pruritus, exertional dyspnea, and dizziness. On initial presentation to the emergency department, she received oral prednisone, topical hydrocortisone, and cetirizine before being discharged. The following day, the patient presented to the emergency department again with chest pain, tachycardia, and diaphoresis. Initially, she was vitally stable, had a blood pressure of 125/62 mm Hg and heart rate of 94 beats per minute, and was afebrile. However, she rapidly decompensated and became hypotensive, with a mean arterial pressure less than 65 mm Hg, requiring a peripheral norepinephrine infusion. A highly sensitive troponin assay administered at the time of the patient’s admission indicated a troponin level of 428 ng/L, which subsequently peaked at 688 ng/L. An urgent electrocadiogram did not demonstrate acute ischemic changes. A transthoracic echocardiogram demonstrated a left ventricular ejection fraction of 45% to 50%. Cardiac catheterization demonstrated classic apical ballooning and hyperdynamic basal wall contraction of the left ventricle without any findings of obstructive coronary artery disease, which was suggestive of TCM (Fig 1). The cardiology team initiated standard TCM treatment with a β-blocker and an angiotensin receptor blocker (ARB).

**Fig 2:**
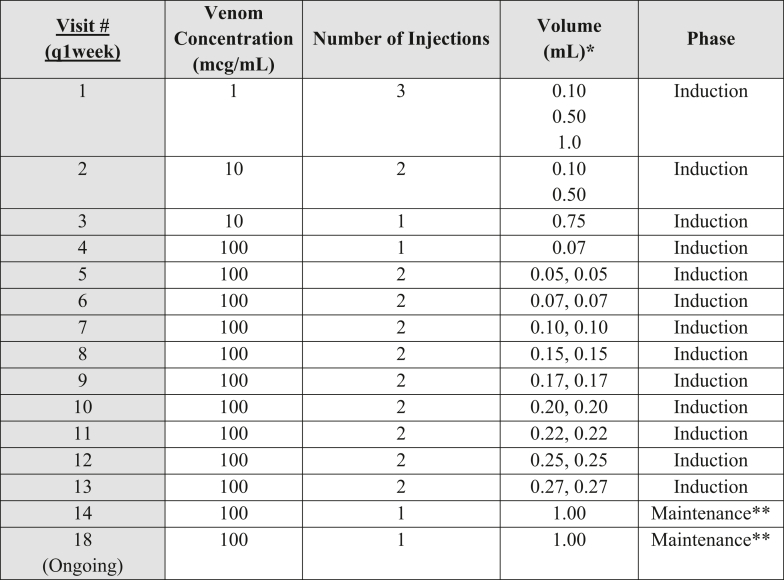
This modified venom desensitization protocol is a modified VIT protocol for patients taking cardiovascular medications, particularly β-blockers. An adapted standard VIT protocol from the Canadian Society Allergy and Clinical Immunology is referenced.^3^**∗**Multiple doses per visit spaced in 15-minute increments with a 30-minute observation period after the final dose. ∗∗The maintenance dose is 1.00 mL administered every 4 weeks for 1 year, after which the interval may extend beyond 4 weeks.

Serum IgE testing demonstrated sensitization to yellow jacket (an IgE antibody level of 29.56 kU/L [normal high level = 0.34 kU/L]) and wasp venom (an IgE antibody level of 2.38 kU/L [normal high level = 0.34 kU/L]). The patient’s basal serum tryptase level was normal, and the she did not have a history of anaphylaxis or urticaria. After a discussion of the risks and benefits of initiation of VIT, the patient was provided with an epinephrine autoinjector. In view of the patient’s increased risk of adverse reactions, we proposed a modified 5-year VIT protocol to develop and maintain desensitization (Fig 2).^3^ The discussions between the patient and her provider included the risk of development of difficult-to-treat anaphylaxis both during VIT and after future venom exposures given her cardiovascular history and concomitant use of β-blockers and an ARB.^4^ She made the informed decision to pursue VIT and opted to carry an epinephrine autoinjector.

During the buildup phase, the patient developed a large local reaction (LLR) that resolved with antihistamines and montelukast. To reduce the risk of recurrent local reactions, we decreased the number of injections given per visit. One year into maintenance VIT, the patient deferred to carry an epinephrine autoinjector after we explained the reduced risk of systemic reaction in a shared decision-making process, as recommended by the 2017 American Academy of Asthma, Allergy & Immunology’s stinging insect hypersensitivity practice parameters.^5^ To date, the patient has successfully completed more than 1 year of VIT at a maintenance dose of 100 μg every 4 weeks.

## Discussion

TCM is characterized by transient dysfunction of the heart and involves temporary hypokinesis, dyskinesis, or akinesis in left ventricular segments with or without apical involvement.^1^ In the setting of allergic reactions, Kounis syndrome (or “allergic angina”), a form of acute coronary syndrome, occurs when anaphylaxis leads to the release of inflammatory mediators such as histamine, leukotrienes, and cytokines, causing coronary artery plaque rupture.^6^ Patients with Kounis syndrome can present with chest pain in the setting of a myocardial infarction secondary to an anaphylactic event.^7^ A retrospective study in South India reported 13 cases of Hymenoptera-induced cardiac manifestations thought to be related to Kounis syndrome.^8^ Generally, a coronary angiogram and cardiac magnetic resonance imaging are helpful to differentiate Kounis syndrome from TCM, as the diagnosis of TCM is supported by the absence of cardiac ischemia, infarction, or active inflammation.^1^ Sequelae of Kounis syndrome and TCM following adrenaline administration together are exceedingly rare and defined as the adrenaline, Takotsubo, anaphylaxis, and Kounis complex, discussion of which is limited to case reports in the literature. The pathophysiology of the adrenaline, Takotsubo, anaphylaxis, and Kounis complex is poorly understood, but a mechanism proposed by Kounis et al suggests a transient coronary spasm or Takotsubo syndrome caused by the inflammatory mediators released during anaphylaxis and the release of endogenous catecholamines subsequent to administration of exogenous adrenaline.^9^

VIT is a highly effective treatment for patients with anaphylaxis in response to Hymenoptera stings, with a systemic reaction rate reduced from 60% to 5% once the patient has reached a maintenance dose of treatment. Treatment duration typically ranges from 3 to 5 years of therapy, but patients may choose to continue treatment indefinitely on the basis of shared decision making between the provider and patient.^2^ VIT involves exposing the patient to increasing doses of the venom of interest. Once the maintenance dose of VIT is reached, patients develop clinical protection against insect venom stings. After 4 or more years of VIT, approximately 75% of patients develop sustained unresponsiveness.^2^ Patient with cardiovascular disease should be judiciously offered VIT as they have a reduced compensatory reserve and are at increased risk of anaphylaxis and mortality.^5^ Importantly, cardiac medications such as β-blockers may reduce the efficacy of epinephrine and exacerbate anaphylactic reactions due to unopposed α-adrenergic stimulation.^5^ VIT protocols vary between centers; however, a modified rush protocol is commonly used to build patients up to a maintenance dose within 6 to 8 weeks.^10^ The Canadian Society Allergy and Clinical Immunology guidelines recommend a 100-μg maintenance dose, which is roughly 5 to 10 times the venom content in a yellow jacket sting.^3^ Given our patient’s risk factors, we pursued a slower 13-week buildup phase with a maintenance dose of 100 μg.

This case posed several clinical dilemmas. First, we asked whether the patient had experienced true anaphylaxis, as her initial presentation was limited to a LLR with delayed cardiac symptoms that resolved without epinephrine administration. Unfortunately, her serum tryptase level was not measured in the emergency department. Multidisciplinary discussions with academic allergists and cardiologists determined that a plausible explanation for her LLR and delayed-onset TCM was a hypersensitivity reaction to an insect sting. The second clinical challenge was weighing the risks and benefits of VIT in a patient with cardiovascular comorbidities (ie, TCM) to whom prescribed an ARB and β-blocker were prescribed.^4^^,^^5^^,^^10^ To mitigate the risks of systemic reaction to VIT, we modified the venom desensitization protocol and allowed the patient to continue taking her cardioprotective medications. The third clinical challenge was related to provision of an epinephrine autoinjector. There are known risks of anaphylaxis and TCM relapse due to catecholamine surge induced by future insect stings and/or epinephrine use. Observational studies have shown an increased risk of systemic reactions with subsequent insect stings in patients who have previously experienced allergic sting reactions and a greater risk in those who have had more severe reactions.^2^ Theoretically, our patient had an LLR, which carries a 10% risk of future systemic reaction and less than a 5% risk of anaphylaxis.^2^ Nonetheless, the patient expressed a great fear of future stings and made an informed decision to pursue VIT and carry an epinephrine autoinjector.

To our knowledge, this is the first case report in Canada of presumed TCM induced by Hymenoptera envenomation. Shared decision making is paramount when considering VIT initiation and prescription of an epinephrine autoinjector in patients with known cardiac sequalae. A modified immunotherapy protocol can be considered to optimize compliance, safety, and efficacy of venom immunotherapy while minimizing the risk of adverse events and reduced efficacy.

Informed consent: Informed consent was obtained from the patient in this study following the principles of the Declaration of Helsinki. Echocardiogram images were retrieved from the St Michael's Hospital Virtual Echo Rounds Library and are publicly accessible via their YouTube channel (available at https://www.youtube.com/@smhecho).

## Disclosure statement

Disclosure of potential conflict of interest: S. Jeimy reports receipt of honoraria from GSK, AbbVie, and AstraZeneca for speaking engagements as well as from GSK, AbbVie, ALK for advisory board or speakers’ bureau membership. The rest of the authors declare that they have no relevant conflicts of interest.
